# Anti-SRP immune-mediated necrotizing myopathy responsive to ofatumumab: a case report

**DOI:** 10.3389/fimmu.2023.1301109

**Published:** 2023-12-19

**Authors:** Sihui Chen, Jing Yang, Du He, Jiajia Fu, Xiaohui Lai, Bi Zhao, Xueping Chen, Huifang Shang

**Affiliations:** ^1^ Department of Neurology, West China Hospital, Sichuan University, Chengdu, Sichuan, China; ^2^ Department of Pathology, West China Hospital, Sichuan University, Chengdu, Sichuan, China

**Keywords:** anti-SRP, immune-mediated necrotizing myopathy, ofatumumab, OFA, case report

## Abstract

**Background:**

Immune-mediated necrotizing myopathies (IMNM) is a rare disease that was first described in 2004. Due to the lack of large case series, there are no formal treatment recommendations for IMNM.

**Methods:**

We presented a case of a 47-year-old woman who experienced progressive limb weakness, starting from the lower limbs and gradually affecting the upper limbs. She also reported experiencing dyspnea after engaging in daily activities. When she was admitted to the hospital, her upper limbs were almost unable to move and she could not stand even with support. Her Creatine kinase (CK) level significantly increased (> 3500 u/l). Electromyography showed myogenic damage, anti-Signal recognition particle (anti-SRP) and anti-Ro52 antibodies were highly positive. Pathological biopsy of the right biceps muscle showed necrotizing myopathy in the skeletal muscle. She was ultimately diagnosed with anti-SRP IMNN, and was given monotherapy with methylprednisolone and combination therapy with immunoglobulin, but her symptoms continued to worsen. The patient refused to bear the possible further liver dysfunction and blood system damage caused by Cyclophosphamide and Rituximab, and she chose to try to use Ofatumumab (OFA).

**Results:**

After receiving three doses of OFA treatment without any adverse reactions, she reported that her muscle strength had basically recovered and she was able to walk independently. The B cells in the circulatory system have been depleted, and blood markers such as liver function have consistently remained within normal range. During the follow up, her activity tolerance continued to improve.

**Discussion:**

We have presented a severe case of SRP-IMNM in which the patient showed poor response to conventional immunotherapy. However, rapid symptom relief was achieved with early sequential use of OFA treatment. This provides a new option for the treatment of SRP-IMNM, and more large-scale studies will be needed in the future to verify our results.

## Case report

A 47-year-old woman presented to the gastroenterology outpatient clinic with elevated levels of alanine aminotransferase (ALT) and aspartate aminotransferase (AST), measuring 211IU/L and 280IU/L respectively. She only presented a slight fatigue, and the laboratory examination showed a high positive Hepatitis A Virus-IgG (HAV-IgG) antibodies and antinuclear antibody (ANA) greater than 1:1000. She has no underlying medical conditions and she had received regular annual physical examination on March 2023. The result of chest CT showed no abnormalities at that time. Therefore, she was diagnosed with AIH despite the lack of liver biopsy evidence. She received the treatment of entecavir 10mg q.d and prednisone 4mg q.d for two months, but her liver function and physical endurance did not show evident improvement. After infected with coronavirus-19 (COVID-19), and her condition rapidly deteriorated, as she began experiencing difficulties in lifting her neck, walking, holding objects with both hands, and even developed dyspnea during moderate activities. When she was admitted, the muscle power of Grade 2 (MMT8 score) proximal, Grade 3 distal muscle strength of the limbs, and Grade 2 the head raising and neck flexion muscle strength without significant muscle atrophy or fasciculation. Tendon reflexes were absent, Babinski’s sign, sensory and autonomic abnormalities were negative.

**Figure 1:**
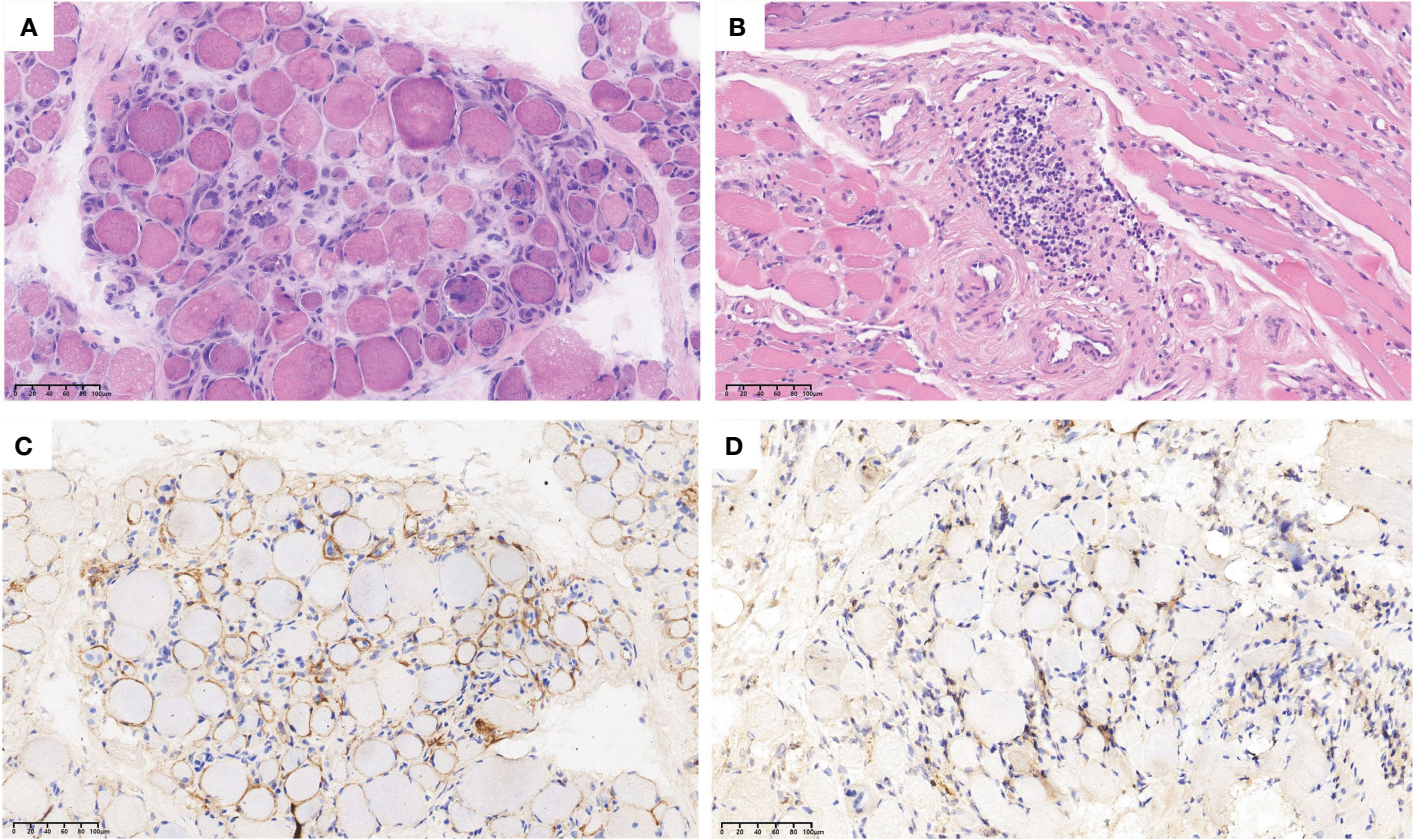
Histopathological and immunohistochemical analysis (H&E) revealed a significant presence of degenerated, necrotic, and regenerating muscle fibers with heterogeneous sizes **(A)**; Around the perimysium of small blood vessels, there were clusters and infiltration of lymphocytes **(B)**; C5b-9 showed strong positivity on the muscle fiber membrane **(C)**; MHC-I was also expressed on the muscle fiber membrane **(D)**.

The laboratory examination revealed a significant increase in levels of creatinine kinase (CK) and myocardial markers (**Table 1**). Electromyography (EMG) showed typical features of myopathic impairments (**Supplementary Material**). Myositis-specific autoantibodies including anti-SRP and anti-Ro52 antibody were high positive (+++). Biopsy of the right biceps brachii muscle showed necrotizing myopathy with minimal local inflammation (**Figure 1**). Echocardiography (ECHO) showed a small amount of pericardial effusion, and electrocardiogram showed abnormal ST-T segment changes. Further examinations did not reveal the presence of an underlying tumor, and the patient denied having any other diseases. She was diagnosed with SRP- IMNM according to the new European Neuromuscular Centre criteria (1).

**Table 1:** Auxiliary examinations in this patient.

Items	*value*	Reference range
CK (u/l)	5437	20-140
LDH (u/l)	958 u/l	120-250
Creatinine (ng/ml)	22	48-79
Hemoglobin (ng/ml)	401.4	115-150
Myoglobin (ng/ml)	824.80	<58.0
CK-MB (ng/ml)	>300.00	<2.88
Troponin-T (ng/ml)	2369.0	0-14
NT-pro-BNP (g/l)	220.0	0-153
ALT (umol/L)	211	<40.0
AST (umol/L)	280	<35.0
Bilirubin (umol/L)	9.0	5.0-28.0
CER (mg/L)	252	210-530
ANA	1:1000	negative
C3 (ng/l)	0.7150	0.758-1.520
C4 (ng/l)	0.1100	0.145-0.360
Ferritin (ng/l)	401.1	15-200
High precision HBV load (IU/ml)	negative	negative
D-dimer (mg/u)	0.74	<0.55
Fibrinogen (g/l)	1.75	2.0-4.0
Tumor markers	negative	negative
IgG4	negative	negative
IgG/A/M	negative	negative
gamma-globulin	negative	negative
Other autoimmune antibodies	negative	negative

CK, Creatine kinase; LDH, lactic dehydrogenase; CK-MB, Creatine kinase isoenzyme; NT-pro-BNP, N-terminal fragment brain natriuretic peptides; ALT, Alanine aminotransferase; AST, Aspartate aminotransferase; CER, Ceruloplasmin; ANA, Antinuclear antibody.

C3, Complement C3; C4, Complement C4; IgG/A/M, Immunoglobulin G/A/M.

The patient was initially treated with methylprednisolone for 5 days (1 g/day), but her muscle weakness became progressively worsened (Grade 1 the head raising and neck flexion muscle strength, Grade 1 proximal and Grade 2 distal muscle strength of the limbs), with dysphagia and more severe dyspnea. She underwent high-resolution chest CT immediately, which revealed mild interstitial changes in her lungs. Then, she received intravenous IVIG (0.4mg/kg/d) for 5 days, but her condition was not attenuated. Sequential immunotherapy with a second round of IVIG, rituximab (RTX), cyclophosphamide and OFA was recommended. However, the patient and her guardians chose OFA but refused other recommendations because they worried about the potential side effects especially the impairment of liver function.

**Figure 2:**
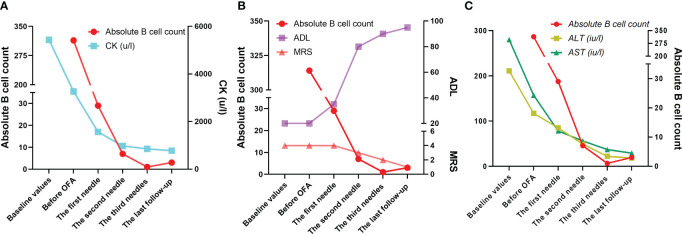
During treatment with OFA, the patient’s CK level declined in line with his absolute B-cell count **(A)**. The MRS scores increased with the decrease of absolute B cell count, but the ADL scores increased significantly, indicating that her ability of daily living was significantly improved **(B)**. ALT and AST levels decreased as the patient’s absolute B-cell count decreased, and both normalized by the second dose of OFA **(C)**. OFA, Ofatumumab; CK, Creatine Kinase; MRS, Modified Rankin Scale; ADL, Activity of Daily Living Scale; ALT, Alanine aminotransferase; AST, Aspartate aminotransferase.

On the second day after the completion of IVIG, she received the first dose of OFA (20 mg) *via* subcutaneous injection. On the second day of first OFA therapy, her muscle strength of limbs and dyspnea were markedly improved (the head raising and neck flexion muscle strength recovered to Grade 2, and distal muscle strength of the limbs recovered to Grade 3), and the absolute B cell counts decreased from 314 to 29 (cell/ul). The patient received the second and third needle of OFA once a month, and the dose for these two injections was 20mg each. Her activity tolerance continued to improve, and B cell remained at a relatively low level during the use of OFA (**Figure 2**). The changes of her clinical symptoms during the treatment are shown in **Table 2**. Myocardial markers, ECG and ECHO returned to normal, but the EMG at two follow-up visits showed that nerve conduction velocities in the extremities were normal but without any improvement in the typical myopathy features (**Supplementary Material**).

**Table 2:** Changes in clinical symptoms during the medical treatment.

		Onset time of treatment	Progressive disease progression	The first injection OFA	The second day OFA	The second injection OFA	The third injection OFA	The last follow-up
	Time	On April 15, 2023	On April 20, 2023	On April 25, 2023	On April 26, 2023	On May 25, 2023	On June 22, 2023	On August 25, 2023
	Medication	Methylprednisolone	IVIG, Prednisone	OFA, Prednisone	OFA, Prednisone	OFA, Prednisone	OFA	OFA
Muscle strength (MMT8)	Proximal left upper limb	2	1	1	1	3	3	3
	Distal left upper limb	3	2	2	3	4	4+	4+
	Proximal right upper limb	2	1	1	1	2+	3	3
	Distal right upper limb	3	2	1	3	4	4+	4+
	Proximal left lower limb	2	1	1	1	3	4	4
	Distal left lower limb	3	2	2	3	4	4	4
	Proximal right lower limb	2	1	1	1	2+	3+	4
	Distal right lower limb	3	2	1	3	4	4	4
	Head raising muscle	2	1	1	2	4	5	5
	Neck flexion muscle	2	1	1	2	4	5	5
Other symptoms	Dyspnea (yes/no)	yes	yes	yes	no	no	no	no
	Dysphagia (yes/no)	no	yes	yes	yes	no	no	no

MMT8, manual muscle test; OFA, Ofatumumab.

## Discussion

The present case was initially diagnosed as AIH. Although both ALT and AST levels can be derived from liver and muscle, ALT is mainly derived from the liver, while AST is primarily derived from the heart and muscle (2). In this patient, the significantly higher AST compared to ALT, without any cardiac involvement, raised concerns about muscle damage. In addition, a recent study found that some IMNM patients may remain asymptomatic for a long time, with only abnormalities in liver enzymes (3). Therefore, abnormal liver function may be a subclinical manifestation in IMNM; CK and EMG examinations are helpful for the diagnosis.

Our patient experienced a significant worsening of clinical symptoms following the COVID-19 infection. Several case reports have been published regarding IMNM associated with COVID-19 infections (4–6). These reports highlighted the potential connection between infections and the exacerbation of IMNM symptoms. COVID-19 may cause IMNM either by infecting muscle fibers directly or by activating the complement system by virus-antibody complexes and viral toxin deposition (7). Interestingly, the patient’s symptoms showed significant improvement after OFA treatment, although there were no significant changes observed in the EMG results. It is possible that the already necrotic muscle cells were too severe to be salvaged or that the follow-up time was not sufficient to observe changes in the EMG. However, the decrease in spontaneous potential does suggest a controlled progression of the acute phase.

Anti-B cell therapy is currently being explored as a potential treatment for IMNM (8). However, when second-line therapies including anti-B cell therapy should be initiated, is still in the exploratory stage. In a study involving 18 IMNM patients treated with RTX, it was observed that more than half of these patients experienced either no or only partial clinical remission. The authors suggested that this outcome may be attributed to the delayed administration of RTX (1). A recent case reported the efficacy of OFA in treating refractory SRP-IMNM (9). Unlike RTX, OFA is a human IgG1 monoclonal antibody against which prevents the risk of antidrug-antibodies production. A previous study has found that the patient who had severe adverse reactions to the infusion of RTX can still be safely and effectively treated with OFA (10). OFA also has the ability to increase B cell binding affinity and enhance complement-dependent cytotoxicity (11). Other studies have also demonstrated the significant therapeutic effects of OFA for conditions resistant to RTX, such as nephrotic syndrome and other systemic autoimmune diseases (12, 13).

We presented a case of severe IMNM in which early administration of OFA was initiated. This patient experienced difficulties with swallowing and breathing as the disease progressed. Despite receiving high-dose methylprednisolone and IVIG treatment, there was no improvement. However, these symptoms rapidly improved following OFA treatment. The rapid clinical improvement observed following OFA treatment and the close correlation between circulating B cell counts and clinical presentation suggest a significant role for OFA in the treatment of SRP-IMNN. In addition, this patient did not report any adverse effects and she has a continued improvement in liver function during the administration of OFA, suggesting the safety and well tolerated of OFA.

Our case is different from the previous case (9). Our patient was treated with OFA the day after the end of IVIG treatment, whereas that case started OFA 10 days after the end of conventional immunotherapy. Our patient seems to have faster clinical remission than that patient. Previous studies have also shown that delayed use of RTX may not be conducive to the improvement of clinical symptoms in patients with IMNM (1). Actually, our study explores the potential of “triple therapy,” incorporating OFA, and proposes that earlier initiation of OFA treatment may offer greater benefits to patients with severe SRP-IMNM. From the mechanism of drug action, IVIG has unique advantages for pre-existing autoimmune antibodies in the circulatory system. IVIG has a broad-spectrum effect against normal human proteins and some special antibodies, allowing it to rapidly neutralize autoimmune antibodies. However, there was no significant effect on pathogenic B cells (12). OFA plays a role mainly through the combination of two unique epitopes on CD20-expressing B cells, inducing the lysis and elimination of pathogenic B cells and reducing the re-production of autoimmune antibodies (13). Therefore, IVIG and OFA may have a synergistic effect in the treatment of IMNM, and earlier sequential use of OFA may provide greater benefit to the severe SRP-IMNM patients.

Although significant efficacy has been observed, we still need further follow-up to clarify the role of OFA in long-term prognosis and recurrence of SRP-IMNN patients.

## Conclusion

Early or asymptomatic anti-SRP-IMNN should be differentiated from liver disease. For severe anti-SRP-INMN patients with a poor response to conventional immunotherapy, early sequential OFA therapy may be an effective, safe, and convenient choice.

## Data availability statement

The original contributions presented in the study are included in the article/**Supplementary Material**. Further inquiries can be directed to the corresponding author.

## Ethics statement

The studies involving humans were approved by Ethics Committee of West China Hospital, Sichuan University. The studies were conducted in accordance with the local legislation and institutional requirements. The participants provided their written informed consent to participate in this study. Written informed consent was obtained from the individual(s) for the publication of any potentially identifiable images or data included in this article.

## Author contributions

SC: Data curation, Investigation, Writing – original draft, Writing – review & editing. JY: Investigation, Resources, Supervision, Writing – review & editing. DH: Data curation, Resources, Supervision, Writing – original draft. JF: Data curation, Writing – review & editing. XL: Data curation, Writing – review & editing. BZ: Data curation, Resources, Writing – review & editing. XC: Resources, Supervision, Writing – review & editing. HS: Data curation, Resources, Supervision, Writing – review & editing.
