# Supplementary material for: Anti-SRP immune-mediated necrotizing myopathy responsive to ofatumumab: a case report
Source: Front Immunol. 2023 Dec 19;14:1301109. doi: 10.3389/fimmu.2023.1301109 (PMC10758405; doi:10.3389/fimmu.2023.1301109)
Supplement: Supplementary file 1 [file Table_1.docx]

| Changes in EMG before and after treatment with OFA (motor nerves) | | | | | | | | | | | |
| --- | --- | --- | --- | --- | --- | --- | --- | --- | --- | --- | --- |
|  | Before using OFA | | | | |  | After using OFA | | | | |
| motor nevers | Lat SD (ms) | Amp SD (mv) | CV SD (m/s) | AMP% SD (%) | F SD (ms) |  | Lat SD (ms) | Amp SD (mv) | CV SD (m/s) | AMP% SD (%) | F SD (ms) |
| Right Medianus |  |  | 59.3 | -3 | 22.8 |  |  |  | 55 | -1 | 25.7 |
| Wrist-APB | 2.8 | 5 |  |  |  |  | 2.8 | 7.3 |  |  |  |
| Bel Elb-Wrist | 5.5 | 4.9 |  |  |  |  | 6.8 | 7.3 |  |  |  |
| Left Medianus | 2.6 | 1.6 |  |  | 20.7 |  | 3.3 | 12.7 |  |  | 26.0 |
| Wrist-APB |  |  |  |  |  |  |  |  |  |  |  |
| Right Ulnaris |  |  |  |  |  |  |  |  |  |  |  |
| Wrist-ADM | 2.2 | 6.7 | 66.7 | -17 | 23.7 |  | 2.1 | 7.0 | 53.6 | -10 | 26.3 |
| Bel Elb-Wrist | 5.2 | 5.6 |  |  |  |  | 6.3 | 6.3 |  |  |  |
| Left Ulnaris | 2.1 | 6.4 |  |  | 22.5 |  | 2.8 | 9.3 |  |  | 25.7 |
| Wrist-ADM |  |  |  |  |  |  |  |  |  |  |  |
| Right Tibialis | 3.6 | 11 |  |  | 44.8 |  | 4.0 | 15.0 |  |  | 47.0 |
| Ankle-AHB |  |  |  |  |  |  |  |  |  |  |  |
| Left Tibialis | 2 | 7.1 |  |  | 42.5 |  | 3.4 | 12.0 |  |  | 44.5 |
| Ankle-AHB |  |  |  |  |  |  |  |  |  |  |  |
| Right Peroneus | 2.8 | 3.4 |  |  | 45.5 |  | 2.8 | 5.0 |  |  | 45.5 |
| Ankle-EDB |  |  |  |  |  |  |  |  |  |  |  |
| Left Peroneus | 2.6 | 3.1 |  |  | 43.3 |  | 3.5 | 4.0 |  |  | 45.0 |
| Ankle-EDB |  |  |  |  |  |  |  |  |  |  |  |

Changes in EMG before and after treatment with OFA (sensory nerves)

| sensory nevers | before OFA treatment | | | | | after OFA treatment | | | | |
| --- | --- | --- | --- | --- | --- | --- | --- | --- | --- | --- |
|  | Lat SD  (ms) | | Amp SD  (mV) | | CV SD  (m/s) | | Lat SD  (ms) | | Amp SD  (uV) | CV SD  (m/s) |
| Right Medianus | 1.92 | 0.4 | 16 | 65.1 | | | 2.3 | 2.6 | 30 | 54.3 |
| Dig III-Wrist |  |  |  |  |  |  |  |  |  |  |
| Left Medianus | 1.83 | -0.1 | 13 | 65.6 | | | 2.2 | 2.0 | 26 | 56.8 |
| Dig III-Wrist |  |  |  |  |  |  |  |  |  |  |
| Right Ulnaris | 1.48 | -3.4 | 9.2 | 64.2 | | | 1.75 | -2.7 | 18 | 60.0 |
| Dig V-Wrist |  |  |  |  |  |  |  |  |  |  |
| Left Ulnaris | 1.52 | -3.3 | 7.8 | 69.1 | | | 1.54 | -3.2 | 17 | 61.7 |
| Dig V-Wrist |  |  |  |  |  |  |  |  |  |  |
| Right Suralis | 2.5 |  | 17 | 56.0 | | | 2 |  | 28 | 55.0 |
| Ankle-Foreleg |  |  |  |  |  |  |  |  |  |  |
| Left Suralis | 1.79 |  | 17 | 58.7 | | | 1.79 |  | 28 | 53.1 |
| Ankle-Foreleg |  |  |  |  |  |  |  |  |  |  |

Changes in EMG before and after treatment with OFA (motor nerves)


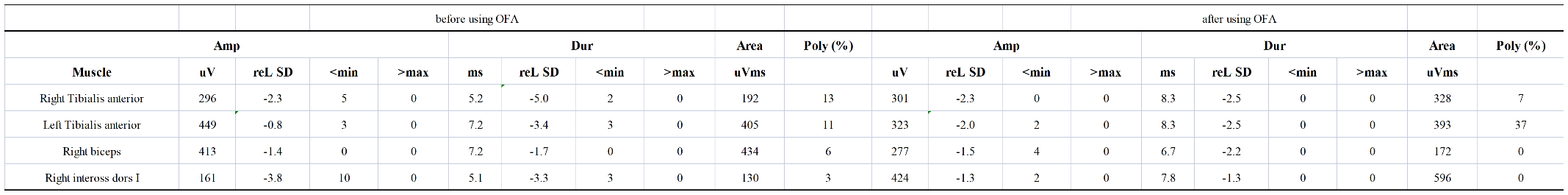


The electromyography (EMG) of this patient before and after treatment showed that the conduction amplitude of the upper and lower limbs decreased, and the upper and lower limbs showed neurogenic damage. Amp: Amplitude; Dur: Duration; Poly%: Percentage of polyphasic waves (%).
